# Partial vs. complete course of antenatal corticosteroid prophylaxis: An Italian single center retrospective study

**DOI:** 10.3389/fped.2022.894526

**Published:** 2022-08-15

**Authors:** Serena Xodo, Giulia Trombetta, Lisa Celante, Carla Pittini, Lorenza Driul, Angelo Cagnacci, Ambrogio P. Londero

**Affiliations:** ^1^Department of Gynecology and Obstetrics, School of Medicine of Udine, Udine, Italy; ^2^Unit of Neonatology, University Hospital of Udine, Udine, Italy; ^3^Department Medical Area, School of Medicine, University of Udine, Udine, Italy; ^4^Academic Unit of Obstetrics and Gynecology, Department of Neuroscience, Rehabilitation, Ophthalmology, Genetics, Maternal and Infant Health, University of Genoa, Genova, Italy

**Keywords:** betamethasone, antenatal corticosteroid prophylaxis, preterm birth, respiratory distress syndrome, intraventricular hemorrhage

## Abstract

**Introduction:**

This study aimed to compare the outcomes of preterm infants given 12 vs. 24mg of betamethasone prophylaxis to understand whether a partial course of antenatal corticosteroids (CCS) could prevent or mitigate the major preterm birth complications.

**Methods:**

This is a retrospective single-center cohort study including neonates born between 24 and 34 weeks of gestation from 2001 to 2019 at the University Hospital of Udine. The study population was divided into two groups: one group received 12mg, and another received a 24mg dose of betamethasone before the delivery. A separate analysis was performed for single and multiple pregnancies. The two groups were evaluated for various neonatal outcomes.

**Results:**

The study population included a total of 1,258 pregnancies and 1,543 neonates delivered between 24 and 34 weeks of gestation, of which 1,022 (803 single and 219 multiple pregnancies) were exposed to the complete CCS prophylaxis, whereas 236 (192 single and 44 multiple pregnancies) received the incomplete CCS prophylaxis. In single pregnancies, as for maternal characteristics, the most significant differences observed between the two groups are the following: a higher prevalence of spontaneous vaginal deliveries in the incomplete CCS prophylaxis (36.46 vs. 23.91%) and, by contrast, a higher prevalence of cesarean deliveries in the complete CCS prophylaxis group (75.72 vs. 63.02%). As for neonatal outcomes, the low Apgar score in the first and fifth min was significantly more prevalent in the incomplete CCS prophylaxis group compared with the complete CCS prophylaxis group. The group of incomplete CCS prophylaxis reported a higher occurrence of the following outcomes: IVH grade 3-4 (7.81 vs. 3.74%, *p* < 0.05), PVL (7.29 vs. 1.99% *p* < 0.05), ROP (23.96 vs. 18.06% *p* = 0.062), and RDS (84.38 vs. 78.83% *p* = 0.085). After adjusting for covariates, the complete CCS prophylaxis group in single pregnancies was significantly protective for IVH grade 3-4, PVL, and low Apgar's scores. Similar results were found in multiple pregnancies except for RDS.

**Discussion:**

This retrospective single-center cohort study found that, compared with preterm infants treated with 24mg betamethasone *in utero*, those given half course of betamethasone had a significantly higher prevalence of IVH grade 3-4, PVL, RDS, and lower Apgar scores at 1 and 5 min. In conclusion, the evidence from this single-center retrospective study supports the preference for the complete CCS prophylaxis in women at risk of preterm birth because of its beneficial effect on the main adverse outcomes.

## Introduction

The ability of corticosteroids (CCS) to induce fetal lung maturation was accidentally demonstrated in 1969 by Liggins while testing the effect of dexamethasone on premature parturition of lambs (1). In 1972 Liggins and Howie finalized the first randomized controlled trial (RCT) in humans, demonstrating the effectiveness of two maternal injections of 12 mg betamethasone given 24 h apart in reducing the incidence of respiratory distress syndrome (RDS) (2). Since then, the administration of either betamethasone or dexamethasone to women at risk of imminent preterm labor has become a milestone in preventing the complications of preterm birth. The most recent Cochrane review on this issue reported a significant decrease in the risk of perinatal death, neonatal death, RDS, and intraventricular hemorrhage (IVH) with CCS given before an anticipated childbirth (3).

Recently, the scientific community raised concerns that glucocorticoid therapy during pregnancy could potentially have adverse long-term effects on infants. Two retrospective studies found that using CCS antenatally increased the risk of neurodevelopmental disorders (4, 5). However, the minimum dose required for the appearance of significant beneficial effects in preterm neonates has not yet been determined. Moreover, preterm birth could be precipitous in some circumstances, thus hampering the completion of the antenatal CCS course. In this scenario, whether or not the complete course of antenatal CCS is as effective as the partial course is still questionable.

Hence, in this current study, we decided to retrospectively compare the outcomes of preterm infants given 12 mg (incomplete CCS prophylaxis) vs. 24 mg (complete CCS prophylaxis) of betamethasone prophylaxis to understand whether a partial course of antenatal CCS could prevent or mitigate the major preterm birth complications.

## Methods

### Study design and setting

This study was a retrospective single-center cohort investigation including women who delivered between January 1, 2001, and December 31, 2019, at the Department of Obstetrics and Gynecology of the University Hospital of Udine. This center is a tertiary-level maternity hospital with a neonatal intensive care unit and advanced medical facilities for the mother and the neonate. The internal review board approved the present study, which followed the dictates of the general authorization produced by the Italian Data Protection Authority in matter of processing data for scientific research. Furthermore, all ethical principles of the Helsinki Declaration were heeded.

### Study participants

**Figure 1:**
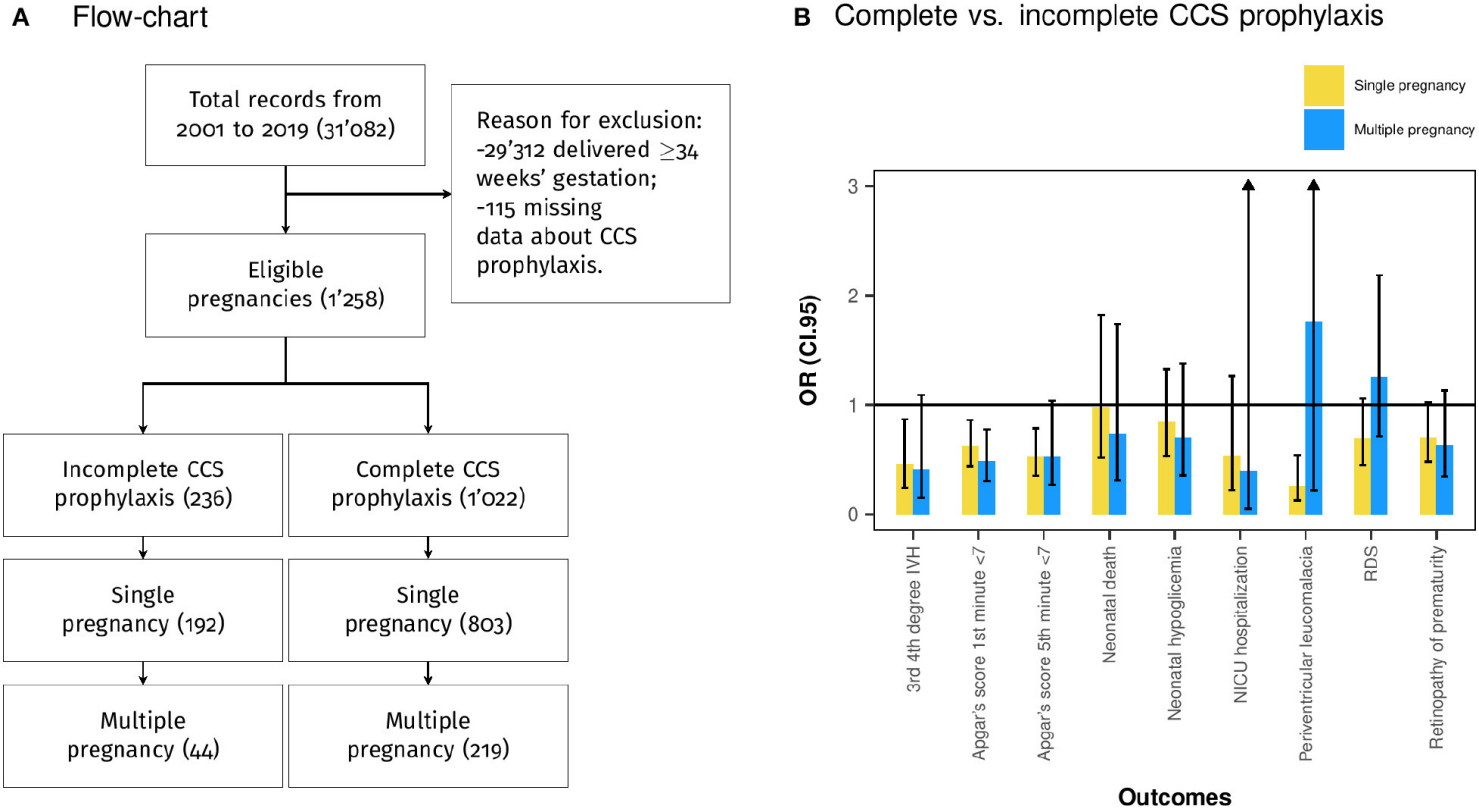
**(A)** Population selection flowchart. **(B)** Bivariate logistic regression analysis considering as independent variable complete corticosteroid (CCS) prophylaxis and dependent variables the assessed outcomes. The plot shows the OR and the 95% confidence interval (CI.95) pairwise for single and multiple pregnancies.

The target population was the total of preterm newborns eligible for CCS prophylaxis. All the consecutive neonates born between 24 and 34 weeks of gestation after preterm labor with intact membranes, preterm premature rupture of membranes and medically indicated delivery were included. The exclusion criteria were gestational age equal to or >34 weeks of gestation and incomplete data about CCS prophylaxis. Population selection is described in Figure 1A.

### Considered variables

The exposure assessed in the present study was the completeness of CCS prophylaxis. Following the aim of the study, the exposure variable was coded into two levels according to the dose of betamethasone administered before delivery: one level represented the group of incomplete CCS prophylaxis (12 mg), and the other level the group of complete CCS prophylaxis (24 mg). The considered outcomes were: Apgar score (at 1st and 5th min), neonatal intensive care unit (NICU) hospitalization, neonatal hypoglycemia, RDS, periventricular leukomalacia (PVL), 3rd 4th-degree IVH, retinopathy of prematurity (ROP), and neonatal death. The covariates were: mother age, parity, mode of conception, pregnancy-related hypertensive disorders (PRHDs), gestational age at delivery, delivery mode, multiple births, neonatal sex, and neonatal weight.

### Definition of outcomes and variables

A low Apgar score was expressed as <7 at the first and fifth min (6). Respiratory distress syndrome was clinically identified by the need for mechanical ventilation and oxygen for at least 48 h and the presence of radiographic chest findings (air bronchograms and reticulogranular appearance of the pulmonary parenchyma). Intraventricular hemorrhage grades were classified according to Papile's classification (7, 8). Moreover, PVL, ROP, and neonatal hypoglycemia were defined as previously described (7, 9, 10). Perinatal death was defined as death occurring <28 days of age (11). PRHDs were classified as previously described, including gestational hypertension, preeclampsia, preeclampsia superimposed on chronic hypertension, and eclampsia (12). The neonatal weight multiple of the median (MoM), also known as birth weight ratio, is the ratio between the observed birth weight and the 50th percentile of birth weight (sex-specific) at the same gestational age (7, 12, 13). Sex-specific neonatal anthropometric charts of the Italian population were used to calculate neonatal weight MoMs (14). Lastly, gestational age was determined by the last menstrual period, confirmed by ultrasound examination during the first and second trimester, and expressed in weeks.

### Data analysis

Data were analyzed through R (version 4.2.0) (15), considering significant a two-tailed *p*-value < 0.05. The statistical analysis was conducted after a Kolmogorov-Smirnoff test application to assess whether the distribution of continuous variables was parametric. An independent analysis was performed for singleton and multiple pregnancies. In singleton pregnancies, where the complete CCS group consisted of 803 pregnancies, a minimum of 33 pregnancies were required in the incomplete CCS group to detect a medium effect size (0.5) in comparing the proportions of the selected outcomes with a power of 80% and significance level of 0.05 (16). In multiple pregnancies, where the complete CCS group consisted of 459 newborns, a minimum of 34 pregnancies were required in the incomplete CCS group to detect a medium effect size (0.5), comparing proportions, with a power of 80% and significance level of 0.05 (16). Bivariate analysis for continuous variables was performed, as appropriate, using the Wilcoxon test (non-parametric variables) or *t*-test (parametric variables). For categorical variables, the differences were tested, as appropriate, with the Chi-square test or Fisher's exact test. Bivariate and multivariate logistic regressions were also performed. The previously selected outcomes were settled as dependent variables. Meanwhile, the exposure and the covariates were settled as independent variables. The initial multivariate model included all possible variables known to be possible confounders or moderators and had a *p* < 0.200 at the bivariate analysis. Interaction terms were maintained in the final multivariate model only if significant, and the NA values were considered missing.

## Results

### Population description

The study population included a total of 1,258 pregnancies and 1,543 neonates delivered between 24 and 34 weeks of gestation, of which 1,022 were exposed to 2 doses of betamethasone 24 h apart (group of complete CCS prophylaxis), whereas 236 received one dose of betamethasone before delivery (group of incomplete CCS prophylaxis) (Figure 1A). In the group of complete CCS prophylaxis 803 single pregnancies and 219 multiple pregnancies were included. Meanwhile, in the group of incomplete CCS prophylaxis 192 single pregnancies and 44 multiple pregnancies (Figure 1A) were enclosed. The median maternal age at delivery was 33.00 years (IQR 29.00–36.00), and the median gestational age at delivery was 31.00 weeks (IQR 28.00–32.00) (Table 1). The population characteristics of singleton and multiple pregnancies are also shown in Table 1.

**Table 1:** Population description.

	**All (1,258)**	**Single pregnancy (995)**	**Multiple pregnancy (263)**
**Patient and pregnancy characteristics**			
Mother age (years)	33 (29–36)	33 (28–36)	33 (29–36)
Nulliparity	62.8% (790/1,258)	60.70% (604/995)	70.72% (186/263)
**Mode of conception**			
Spontaneous	89.98% (1,132/1,258)	95.88% (954/995)	67.68% (178/263)
Ovulation induction/IUI	1.99% (25/1,258)	0.80% (8/995)	6.46% (17/263)
IVF/ICSI	8.03% (101/1,258)	3.32% (33/995)	25.86% (68/263)
**Multiple births**			
Single pregnancy	79.09% (995/1,258)	100.00% (995/995)	
Twins	19.71% (248/1,258)		94.30% (248/263)
Other multiple births	1.19% (15/1,258)		5.70% (15/263)
**Mode of delivery**			
Spontaneous vaginal delivery	22.02% (277/1,258)	26.33% (262/995)	5.70% (15/263)
Operative vaginal delivery	0.32% (4/1,258)	0.40% (4/995)	0.00% (0/263)
Cesarean delivery	77.66% (977/1,258)	73.27% (729/995)	94.30% (248/263)
PRHDs	17.56% (216/1,230)	20.39% (198/971)	6.95% (18/259)
**Neonatal characteristics**			
Neonatal male sex	53.79% (830/1,543)	53.07% (528/995)	55.11% (302/548)
Gestational age (weeks)	31 (28–32)	30 (28–32)	31 (29–32)
Neonatal weight (grams)	1,430 (1,030–1,792)	1,350 (972–1,773)	1,556 (1,186–1,808)
Neonatal weight (MoM)	1 (0.87–1.12)	1.00 (0.85–1.15)	1.00 (0.89–1.09)
Complete CCS prophylaxis	81.79% (1,262/1,543)	80.70% (803/995)	83.76% (459/548)
**Neonatal outcomes**			
Apgar's score 1st min	6 (5–7)	6 (5–7)	7 (5–7)
Apgar's score 5th min	8 (7–8)	8 (7–8)	8 (7–8)
Apgar's score 1st min <7	55.54% (857/1,543)	59.70% (594/995)	47.99% (263/548)
Apgar's score 5th min <7	12.44% (192/1,543)	14.17% (141/995)	9.31% (51/548)
NICU hospitalization	95.72% (1,477/1,543)	94.77% (943/995)	97.45% (534/548)
Neonatal hypoglicemia	11.73% (181/1,543)	12.46% (124/995)	10.40% (57/548)
RDS	80.49% (1,242/1,543)	79.90% (795/995)	81.57% (447/548)
Periventricular leucomalacia	2.59% (40/1,543)	3.02% (30/995)	1.82% (10/548)
3rd 4th degree IVH	4.15% (64/1,543)	4.52% (45/995)	3.47% (19/548)
Retinopathy of prematurity	17.3% (267/1,543)	19.20% (191/995)	13.87% (76/548)
Neonatal death	6.48% (100/1,543)	6.63% (66/995)	6.20% (34/548)

Data are presented as median and interquartile range (IQR), means (±standard deviation), or percentages and absolute values (events/exposed). The NA values are not counted in the exposed denominator. IUI, intrauterine insemination; IVF, in vitro fertilization; ICSI, intracytoplasmic sperm injection; MoM, multiple of the median; CCS, corticosteroids; NICU, neonatal intensive care unit; RDS, respiratory distress syndrome; IVH, intraventricular hemorrhage.

### Singleton pregnancies

Table 2 shows the differences between the complete and incomplete CCS prophylaxis among singleton pregnancies. As for maternal outcomes, the most significant differences observed between the two groups are the following: a higher prevalence of spontaneous vaginal deliveries in the incomplete CCS prophylaxis (36.46 vs. 23.91%) and, by contrast, a higher prevalence of cesarean deliveries (CD) in the complete CCS prophylaxis group (75.72 vs. 63.02%). As for neonatal characteristics, the median birthweight was 1,355 g (IQR 988-1770) in the complete CCS prophylaxis group vs. 1,299 g (IQR 941-1780) in the incomplete CCS prophylaxis group (*p* = 0.611). The median Apgar score at the first min was significantly lower in the incomplete CCS prophylaxis group compared with the complete CCS prophylaxis group (Table 2). And a high prevalence of low pH at the fifth min was present in the incomplete than in the complete CCS prophylaxis group (21.35 vs. 12.45%, *p* < 0.05). The group of incomplete CCS prophylaxis reported a higher occurrence of the following outcomes: IVH grade 3–4 (7.81 vs. 3.74%, *p* < 0.05), PVL (7.29 vs. 1.99% *p* < 0.05), ROP (23.96 vs. 18.06% *p* = 0.062), and RDS (84.38 vs. 78.83% *p* = 0.085) (Table 2). No significant differences were retrieved for the following outcomes: admission to neonatal intensive unit care, the necessity of neonatal cardiopulmonary reanimation, and neonatal death. Table 3 shows the bivariate and multivariate logistic regression analysis. After adjusting for covariates, the complete CCS prophylaxis group was significantly protective for IVH grade 3–4, PVL, low Apgar's scores, ROP, and RDS.

**Table 2:** Population description of singleton pregnancies stratified by complete or incomplete corticosteroid (CCS) prophylaxis.

	**Incomplete CCS prophylaxis (192)**	**Complete CCS prophylaxis (803)**	***p*** **-value**
**Patient and pregnancy characteristics**			
Mother age (years)	32 (28–36)	33 (28–36)	0.498
Nulliparity	63.02% (121/192)	60.15% (483/803)	0.464
**Mode of conception**			0.297
Spontaneous	97.92% (188/192)	95.39% (766/803)	
Ovulation induction/IUI	0.52% (1/192)	0.87% (7/803)	
IVF/ICSI	1.56% (3/192)	3.74% (30/803)	
**Mode of delivery**			<0.05
Spontaneous vaginal delivery	36.46% (70/192)	23.91% (192/803)	
Operative vaginal delivery	0.52% (1/192)	0.37% (3/803)	
Cesarean delivery	63.02% (121/192)	75.72% (608/803)	
PRHDs	16.04% (30/187)	21.43% (168/784)	0.100
**Neonatal characteristics**			
Neonatal male sex	57.81% (111/192)	51.93% (417/803)	0.142
Gestational age (weeks)	30 (28–32)	30 (28–32)	0.365
Neonatal weight (grams)	1,299 (941–1,780)	1,355 (988–1,770)	0.611
Neonatal weight (MoM)	1.01 (0.86–1.16)	1.00 (0.85–1.14)	0.784
**Neonatal outcomes**			
Apgar's score 1st min	5 (4–7)	6 (5–7)	<0.05
Apgar's score 5th min	8 (7–8)	8 (7–8)	<0.05
Apgar's score 1st min <7	68.75% (132/192)	57.53% (462/803)	<0.05
Apgar's score 5th min <7	21.35% (41/192)	12.45% (100/803)	<0.05
NICU hospitalization	96.88% (186/192)	94.27% (757/803)	0.145
Neonatal hypoglycemia	14.06% (27/192)	12.08% (97/803)	0.455
RDS	84.38% (162/192)	78.83% (633/803)	0.085
Periventricular leukomalacia	7.29% (14/192)	1.99% (16/803)	<0.05
3rd 4th degree IVH	7.81% (15/192)	3.74% (30/803)	<0.05
Retinopathy of prematurity	23.96% (46/192)	18.06% (145/803)	0.062
Neonatal death	6.77% (13/192)	6.60% (53/803)	0.932

Data are presented as median and interquartile range (IQR), means (±standard deviation), or percentage and absolute values (events/exposed). The NA values are not counted in the exposed denominator. The p-values refer as appropriate to Wicoxon test, Chi-square test, or Fisher's exact test. IUI, intrauterine insemination; IVF, in vitro fertilization; ICSI, intracytoplasmic sperm injection; MoM, multiple of the median; CCS, corticosteroids; NICU, neonatal intensive care unit; RDS, respiratory distress syndrome; IVH, intraventricular hemorrhage.

**Table 3:** Logistic regression analysis of the considered outcomes in the singleton pregnancies.

	**OR (CI.95)**	***p*** **-value**	OR (CI.95)^*^	p^*^ **-value**
**3rd 4th degree IVH**				
Spontaneous conception ^†^	0.42 (0.14–1.22)	0.110	0.35 (0.1–1.17)	0.087
Neonatal male sex^†^	1.01 (0.56–1.84)	0.971		
CD^†^	0.89 (0.46–1.73)	0.738		
Complete CCS prophylaxis^†^	0.46 (0.24–0.87)	<0.05	0.42 (0.21–0.84)	<0.05
Gestational age (weeks)^‡^	0.63 (0.55–0.71)	<0.05	0.63 (0.55–0.72)	<0.05
Neonatal weight (MoM)^‡^	4.63 (1.14–18.8)	<0.05	3.3 (0.75–14.6)	0.115
**Periventricular leukomalacia**
Spontaneous conception^†^	0.59 (0.14–2.56)	0.481		
Neonatal male sex^†^	2.49 (1.1–5.66)	<0.05	2.39 (1.05–5.46)	<0.05
CD^†^	1.00 (0.44–2.28)	0.993		
Complete CCS prophylaxis^†^	0.26 (0.12–0.54)	<0.05	0.27 (0.13–0.57)	<0.05
Gestational age (weeks)^‡^	0.91 (0.8–1.04)	0.156	0.91 (0.80–1.04)	0.168
Neonatal weight (MoM)^‡^	1.32 (0.25–7.02)	0.745		
**Retinopathy of prematurity**				
Spontaneous conception^†^	2.26 (0.79–6.4)	0.127	3.69 (1.12–12.17)	<0.05
Neonatal male sex^†^	0.87 (0.63–1.19)	0.388		
CD^†^	1.42 (0.97–2.08)	0.068	2.39 (1.48–3.85)	<0.05
Complete CCS prophylaxis^†^	0.7 (0.48–1.02)	0.063	0.60 (0.38–0.95)	<0.05
Gestational age (weeks)^‡^	0.6 (0.56–0.65)	<0.05	0.58 (0.54–0.63)	<0.05
Neonatal weight (MoM)^‡^	0.68 (0.32–1.41)	0.295		
**Apgar's score 1st min <7**				
Spontaneous conception^†^	1.76 (0.94–3.29)	0.078	2.44 (1.18–5.01)	<0.05
Neonatal male sex^†^	0.9 (0.7–1.16)	0.421		
CD^†^	1.62 (1.22–2.14)	<0.05	1.55 (1.1–2.18)	<0.05
Complete CCS prophylaxis^†^	0.62 (0.44–0.86)	<0.05	0.56 (0.38–0.81)	<0.05
Gestational age (weeks)^‡^	0.66 (0.62–0.71)	<0.05	0.65 (0.61–0.70)	<0.05
Neonatal weight (MoM)^‡^	0.5 (0.28–0.91)	<0.05	0.36 (0.18–0.73)	<0.05
**Apgar's score 5th min <7**				
Spontaneous conception^†^	0.79 (0.34–1.83)	0.587		
Neonatal male sex^†^	1.8 (1.24–2.61)	<0.05	1.97 (1.31–2.96)	<0.05
CD^†^	0.77 (0.53–1.14)	0.196	0.97 (0.62–1.52)	0.892
Complete CCS prophylaxis^†^	0.52 (0.35–0.78)	<0.05	0.50 (0.32–0.79)	<0.05
Gestational age (weeks)^‡^	0.66 (0.61–0.71)	<0.05	0.66 (0.61–0.71)	<0.05
Neonatal weight (MoM)^‡^	0.95 (0.42–2.18)	0.907		
**RDS**				
Spontaneous conception^†^	2.14 (1.1–4.16)	<0.05	2.36 (1.17–4.78)	<0.05
Neonatal male sex^†^	0.95 (0.7–1.3)	0.767		
CD^†^	2.12 (1.53–2.95)	<0.05	2.54 (1.76–3.66)	<0.05
Complete CCS prophylaxis^†^	0.69 (0.45–1.05)	0.086	4.7e-5 (1e-8–0.19)	<0.05
Gestational age (weeks)^‡^	0.78 (0.72–0.84)	<0.05	0.60 (0.46–0.77)	<0.05
Neonatal weight (MoM)^‡^	1.95 (0.95–4)	0.069	2.91 (1.29–6.59)	<0.05
Interaction term = complete CCS prophylaxis : gestational age (weeks)^§^			1.36 (1.04–1.77)	<0.05

Bivariate and multivariate (*) models.

Legend:

† These variables are Boolean and are represented in terms of presence vs. absence of a specific characteristic (e.g., complete CCS vs. incomplete CCS prophylaxis).

‡ These are continuous variables [e.g., for gestational age an OR of 0.63 means that for every increased week, we have a reduction of the odds (hence as the variable increases, the event is less likely to occur)].

§ This is an interaction term between a nominal and a continuous variable. The nominal variable is coded as present (1) or absent (0). In this specific case the gestational age has an OR 0.60 and the odds of having RDS decreases by a factor of 0.60 per every additional gestational week if the CCS prophylaxis is incomplete. However, if the prophylaxis is complete the odds of having RDS decreases by a factor of 0.82 (0.60 × 1.36) per every additional week. IVH, intraventricular hemorrhage; CD, cesarean delivery; CCS, corticosteroids; MoM, multiple of the median; NICU, neonatal intensive care unit; RDS, respiratory distress syndrome.

### Multiple pregnancies

Table 1 shows the characteristics of the multiple pregnancies sub-group, and Table 4 shows the differences between complete and incomplete CCS prophylaxis. In addition, Figure 1B shows pairwise the bivariate logistic regression of singleton and multiple pregnancies. The effect of complete CCS prophylaxis is similar in single and multiple pregnancies except for RDS. Furthermore, complete CCS prophylaxis was associated with lower prevalence of low Apgar score at first (*p* < 0.05) and fifth (*p* = 0.060) min, and 3rd 4th-degree IVH (*p* = 0.065) (Table 4).

**Table 4:** Population description of multiple pregnancies stratified by complete or incomplete corticosteroid (CCS) prophylaxis.

	**Incomplete CCS prophylaxis (44)**	**Complete CCS prophylaxis (219)**	***p-*** **value**
**Patient and pregnancy characteristics**			
Mother age (years)	32 (29–37)	33 (30–36)	0.864
Nulliparity	68.18% (30/44)	71.23% (156/219)	0.685
Mode of conception			0.139
Spontaneous	63.64% (28/44)	68.49% (150/219)	
Ovulation induction/IUI	13.64% (6/44)	5.02% (11/219)	
IVF/ICSI	22.73% (10/44)	26.48% (58/219)	
Mode of delivery			0.722
Spontaneous vaginal delivery	6.82% (3/44)	5.48% (12/219)	
Operative vaginal delivery	0% (0/44)	0% (0/219)	
Cesarean delivery	93.18% (41/44)	94.52% (207/219)	
PRHDs	2.38% (1/42)	7.83% (17/217)	0.203
**Neonatal characteristics**			
Neonatal male sex	57.30% (51/89)	54.68% (251/459)	0.649
Gestational age (weeks)	30 (27–32)	32 (29–32)	<0.05
Neonatal weight (g)	1540 (1,152–1,746)	1558 (1,189–1,824)	0.197
Neonatal weight (MoM)	1.02 (0.91–1.13)	1.00 (0.89–1.09)	0.100
**Neonatal outcomes**			
Apgar's score 1st min	6 (5–7)	7 (5–7)	<0.05
Apgar's score 5th min	8 (7–8)	8 (8–9)	<0.05
Apgar's score 1st min <7	62.92% (56/89)	45.10% (207/459)	<0.05
Apgar's score 5th min <7	14.61% (13/89)	8.28% (38/459)	0.060
NICU hospitalization	98.88% (88/89)	97.17% (446/459)	0.350
Neonatal hypoglicemia	13.48% (12/89)	9.80% (45/459)	0.298
RDS	78.65% (70/89)	82.14% (377/459)	0.438
Periventricular leucomalacia	1.12% (1/89)	1.96% (9/459)	0.589
3rd 4th degree IVH	6.74% (6/89)	2.83% (13/459)	0.065
Retinopathy of prematurity	19.10% (17/89)	12.85% (59/459)	0.119
Neonatal death	7.87% (7/89)	5.88% (27/459)	0.478

Data are presented as median and interquartile range (IQR), means (±standard deviation), or percentage and absolute values (events/exposed). The NA values are not counted in the exposed denominator. The p-values refers as appropriate to Wicoxon test, Chi-square test, or Fisher's exact test. IUI, intrauterine insemination; IVF, in vitro fertilization; ICSI, intracytoplasmic sperm injection; MoM, multiple of the median; CCS, corticosteroids; NICU, neonatal intensive care unit; RDS, respiratory distress syndrome; IVH, intraventricular hemorrhage.

## Discussion

### Principal findings

This retrospective single-center cohort study found that, compared with preterm infants treated with 24 mg betamethasone *in utero*, in singleton pregnancies, those given half-courses of betamethasone had a significantly higher prevalence of low Apgar scores at first and fifth min, IVH grade 3–4, PVL, and RDS. Similar results were observed in multiple pregnancies except for RDS. Moreover, spontaneous vaginal delivery occurred more frequently in the group of incomplete CCS prophylaxis, whereas CD was significantly higher in the group of complete CCS prophylaxis.

### Results in the context of what is known

The original regimen used by Liggins and Howie in their first RCT was a mixture of betamethasone phosphate 6 mg and betamethasone acetate 6 mg in 2 doses given 24 h apart (2). The phosphate preparation guarantees rapid exposure to the drug, while the acetate form offers a more sustained exposure (17). This regimen raises the 75% occupancy level of glucocorticoid receptors, providing near-maximal induction of associated genes in fetal tissues. The Authors found in another clinical trial that doubling the dose of betamethasone did not further improve the beneficial effects in preterm neonates (17). From then on, CCS has become the mainstay of prophylactic treatment in preterm birth.

Only a few studies in the literature explored the effects of different dose regimens of antenatal CCS prophylaxis, yielding conflicting results. Studies comparing half course of CCS with no CCS therapy (18–20), showed that the exposure to an incomplete course of prophylaxis decreases the rate of severe neonatal outcomes, especially at very early gestational weeks (25–27 weeks) and in non-tertiary hospitals (18). Other observational studies compared the administration of an incomplete CCS course vs. a complete CCS course (21–24) demonstrating a significant improvement of the neonatal clinical outcome with the half dose exposure. However, a discrepancy between the studies is seen for severe IVH since a significantly lower rate of severe IVH was observed in the incomplete antenatal steroids group in the oldest study (21). At the same time, this trend was not confirmed in the most recent studies (23, 24). The impact of the incomplete course of CCS prophylaxis on severe IVH rate has still to be clarified. Our results suggest that, compared with complete exposure to CCS, an incomplete exposure leads to a significantly higher rate of this unfavorable outcome. Antenatal steroid therapy has been shown to decrease the vulnerability of the germinal matrix vasculature to hemorrhage (25). It could be speculated that the steroid-induced molecular and morphological changes are dose-dependent, thus explaining why compared with the incomplete course of CCS, the complete course significantly lowers the IVH rate. According to our study, the complete CCS prophylaxis significantly decreased the prevalence of PVL, irrespective of the gestational age, as shown in the multivariate regression analysis. It has been postulated that CCS might suppress the upregulation of inflammatory cytokines, thus preventing the injury of preoligodendrocytes through the activation of microglia within the immature white matter (26). Again, the superiority of the complete CCS course over the incomplete one in reducing PVL prevalence may suggest a dose-dependent effect of CCS action.

Surprisingly, our data show that half the standard regimen given to patients with threatened preterm labor might be sufficient to gain benefits on respiratory outcomes in twin pregnancies. A recent metanalysis of 16 observational studies has shown that antenatal CCS may be beneficial in reducing the risk of RDS, mortality, IVH and PVL among preterm twin pregnancies. A sub-analysis found that subjects exposed to the complete course of steroid prophylaxis had a lower OR of RDS (27). However, our study may suggest that fetal lung maturation might be accelerated in twin pregnancies compared to singleton pregnancies. Historically, fetal lung maturity has been extensively investigated, arising conflicting results (27–30). It might be speculated that the accelerated pulmonary maturity in twins could be due to the antenatal competitive environment to which twins are exposed. Since maternal resources have to be shared in multiple pregnancies, twin fetuses respond with a slower rate of fetal growth during the third trimester, and possibly reaching the lung maturation earlier when compared with singleton fetuses.

Additionally, our study revealed that neonates treated with the incomplete CCS prophylaxis had significantly lower mean Apgar scores in the first and fifth min. Both antenatal and postnatal factors are involved in the assignment of a low Apgar score. The score may reflect an injury that occurred before birth or the adequacy of resuscitation. Moreover, Apgar score's effectiveness has been questioned in preterm neonates since their scores could be depressed by their immaturity (31). Interestingly, even after adjustment for gestational age, the Apgar score at the first and fifth min was significantly lower in the group of incomplete CCS prophylaxis, thus hinting at a potential protective role of CCS.

The higher prevalence of spontaneous vaginal deliveries in the group of incomplete CCS prophylaxis in our population reflects the rampage of preterm labor in those cases, leaving no time for the second drug injection. By contrast, the group of complete CCS prophylaxis experienced a higher prevalence of cesarean births, suggesting that they mainly account for iatrogenic preterm births. Compared to vaginal deliveries, CDs are undeservedly thought to be less traumatic for very premature infants because of the avoidance of prolonged labor. A growing body of evidence in the literature shows that compared to vaginal delivery, the CD is not protective in the peri-viable period. For example, a retrospective cohort study on 271 singleton pregnancies delivered between 22 to 29 weeks revealed that CD performed for standard obstetrical indications is associated with an increase in immediate survival, but it is not associated with an overall decrease in morbidity or mortality (32). Another study found no differences in neurodevelopment at 2 years of age in 158 neonates delivered through vaginal delivery and CD at the time of peri-viability (33). Our data confirm that CD was not protective against severe neonatal outcomes such as IVH. In addition, we found CD to be associated in multivariate logistic regression with increased odds of ROP, low Apgar's score at the first min, and RDS.

### Strengths and limitations

The retrospective design of our study is its main limitation. Second, there is an inherent danger in extrapolating conclusions from a cohort study that spans over a decade, given that significant advances in neonatal care have been established over the last few decades, thus hampering the generalizability of our findings. The main strength of this study is the topic considered in light of the scantiness of data in the literature. Another strength of this study is the sample size considered, which is higher than the number of patients included in other retrospective studies.

### Clinical implications

Our study might have a relevant clinical implication. In light of our results, women with threatened preterm birth should be counseled on the superiority of the complete course of CCS over the incomplete one in preventing the major complications related to preterm birth. Additionally, women with twin pregnancy and an imminent delivery should be informed that half a course of antenatal CCS might potentially improve the respiratory outcomes of preterm babies.

### Research implications

Further data would be required to clarify whether half the course of antenatal CCS is sufficient to improve the respiratory outcomes in twin pregnancies, as suggested by our study. If these findings would be confirmed it could be interesting to realize which is the physiological explanation behind this observation.

## Conclusion

In conclusion, the evidence from this single-center retrospective study supports the preference for the complete CCS prophylaxis in women at risk of preterm birth because of its beneficial effect on the main adverse outcomes.

## Data availability statement

The data analyzed in this study is subject to the following licenses/restrictions: The data that support the findings of this study are available, but restrictions apply to the availability of these data. These data were used under license for the current study, and so are not publicly available. However, data are available upon reasonable request to the authors and with permission of the Internal Review Board. Requests to access these datasets should be directed to SX, serenaxodo@yahoo.it.

## Ethics statement

The studies involving human participants were reviewed and approved by the Internal Review Board of the Department of Medical Area (University of Udine). The present study was conducted in accordance with Helsinki Declaration and it followed the dictates of the general authorization to process personal data for scientific research purposes by the Italian Data Protection Authority. The need for an informed consent, according with national legislation, was waived by the IRB listed above because this was a retrospective cohort study. Written informed consent for participation was not required for this study in accordance with the national legislation and the institutional requirements.

## Author contributions

Substantial contributions to conception and design or acquisition of data or to analysis and interpretation of data: SX, GT, LC, CP, LD, AC, and APL. Drafting the article or revising it critically for important intellectual content: SX, CP, LD, AC, and APL. All authors have read and approved the final manuscript.

## Conflict of interest

The authors declare that the research was conducted in the absence of any commercial or financial relationships that could be construed as a potential conflict of interest.

## Publisher's note

All claims expressed in this article are solely those of the authors and do not necessarily represent those of their affiliated organizations, or those of the publisher, the editors and the reviewers. Any product that may be evaluated in this article, or claim that may be made by its manufacturer, is not guaranteed or endorsed by the publisher.

## Abbreviations

CCS: corticosteroids
CD: cesarean delivery
CI.95: 95% confidence interval
ICSI: intracytoplasmic sperm injection
IQR: interquartile range
IUI: intrauterine insemination
IVF: *in vitro* fertilization
IVH: intraventricular hemorrhage
MoM: multiple of the median
NICU: neonatal intensive care unit
OR: odds ratio
PRHD: pregnancy-related hypertensive disorder
PVL: periventricular leucomalacia
RCT: randomized controlled trial
RDS: respiratory distress syndrome
ROP: retinopathy of prematurity.
